# High-Resolution Single-Cell Sequencing of Malaria Parasites

**DOI:** 10.1093/gbe/evx256

**Published:** 2017-12-06

**Authors:** Simon G Trevino, Standwell C Nkhoma, Shalini Nair, Benjamin J Daniel, Karla Moncada, Stanley Khoswe, Rachel L Banda, François Nosten, Ian H Cheeseman

**Affiliations:** Genetics Department, Texas Biomedical Research Institute, San Antonio, Texas; Malawi-Wellcome-Liverpool-Wellcome Trust Clinical Research Programme, Chichiri, Blantyre, Malawi; Liverpool School of Tropical Medicine, Liverpool, United Kingdom; Wellcome Trust Liverpool Glasgow Centre for Global Health Research, Liverpool, United Kingdom; University of Texas Health Science Center at San Antonio, San Antonio, Texas; Shoklo Malaria Research Unit, Mahidol-Oxford Tropical Medicine Research Unit, Faculty of Tropical Medicine, Mahidol University, Mae Sot, Tak, Thailand; Nuffield Department of Medicine, Centre for Tropical Medicine, University of Oxford, United Kingdom

**Keywords:** malaria, single-cell genomics, methods

## Abstract

Single-cell genomics is a powerful tool for determining the genetic architecture of complex communities of unicellular organisms. In areas of high transmission, malaria patients are often challenged by the activities of multiple *Plasmodium falciparum* lineages, which can potentiate pathology, spread drug resistance loci, and also complicate most genetic analysis. Single-cell sequencing of *P. falciparum* would be key to understanding infection complexity, though efforts are hampered by the extreme nucleotide composition of its genome (∼80% AT-rich). To counter the low coverage achieved in previous studies, we targeted DNA-rich late-stage parasites by Fluorescence-Activated Cell Sorting and whole genome sequencing. Our method routinely generates accurate, near-complete capture of the 23 Mb *P. falciparum* genome (mean breadth of coverage 90.7%) at high efficiency. Data from 48 single-cell genomes derived from a polyclonal infection sampled in Chikhwawa, Malawi allowed for unambiguous determination of haplotype diversity and recent meiotic events, information that will aid public health efforts.

## Introduction

Single-cell genomics has helped unravel the population dynamics of unicellular organisms (Blake et al. 2015; Gawad et al. 2016; Nair et al. 2014; Wang and Song 2017), cancer cells (Navin et al. 2011), and developmental lineages (Lodato et al. 2015) in multicellular organisms. Efforts to eradicate malaria, of which nearly half of the human population is at risk, can greatly benefit from understanding the genetic strategies that enable *Plasmodium falciparum* communities to persist. For instance, genome sequencing has played a pivotal role in understanding the spread of drug-resistant parasites (Miotto et al. 2015), global structuring of parasite populations (Manske et al. 2012), and selection of vaccine candidate loci (Amambua-Ngwa et al. 2012). In locations where malaria is endemic, patients are often infected with multiple parasite lineages (Conway et al. 1991; Conway and McBride 1991; Nkhoma et al. 2012, 2013; Snounou et al. 1993). Fundamental details about the individual malaria infections, such as the number of parasite lineages, their diversity and relationship to one another, could be addressed by haplotype reconstruction from individual cells.

Current methods to understand the complexity of malaria infections rely on inferences from either PCR genotyping or whole genome sequencing (WGS; Assefa et al. 2014; Galinsky et al. 2015; Hill and Babiker 1995; Juliano et al. 2010; Manske et al. 2012; O'Brien et al. 2016; Pearson et al. 2016). While these approaches are scalable, affordable, and can provide estimates of key demographic parameters they are generally reliant on assumptions such as random mating (Hill and Babiker 1995), which are frequently violated in parasite populations. Consequently, direct methods to understand the complexity of infections can add considerable depth and accuracy as has been the case for direct phasing methods of human genomes (Song et al. 2017).

The 23 Mb *P. falciparum* genome is a notoriously challenging target for genomics given it is the most AT-rich genome sequenced to date (>80% AT; Gardner et al. 2002), contains a high density of microsatellite repeats and is protected by multiple cell membranes. Culture-based dilution cloning (Rosario 1981) and single-cell genomics (Nair et al. 2014) can be used to isolate individual malaria haplotypes. Although dilution cloning can generate high-quality data from clonal expansion of infected red blood cells (iRBCs; Nkhoma et al. 2012), it is labor intensive, prone to contamination and reliant on parasites to thrive in culture, making it inappropriate for large-scale experiments. To remedy these shortcomings, we previously developed a single-cell genomics approach based on Fluorescence-Activated Cell Sorting (FACS) and whole genome amplification (WGA; Nair et al. 2014). However, a high rate of allelic and genomic dropout limited the analyzable proportion of the genome to <50% and required costly quality control prior to library preparation and WGS.

Recent WGA studies of human nuclei (Leung et al. 2015) and bulk *P. falciparum* DNA (Oyola et al. 2016) suggest that amplification might be improved in reactions containing multiple genome copies. During the ∼48 h life cycle of *P. falciparum* in the blood, late-stage iRBCs generate an average of 16 clonal copies of the parasite’s genome by DNA replication (Reilly et al. 2007). We hypothesized that these DNA-rich parasites, which contain multiple genome templates, would improve WGA reactions and subsequent WGS data quality.

We found that in an asynchronous culture of a well-characterized *P. falciparum* laboratory line, HB3, cells with the highest DNA content yield near-complete genome coverage (mean 92.4%). After optimizing our protocol in clinical samples, we interrogated a polyclonal infection (MAW0) from Chikhwawa, Malawi and recovered similarly high genome coverage (mean 90.7%) for 48 out of 48 attempted reactions. These data allow fine scale estimation of diversity and relatedness within a single malaria infection.

## Materials and Methods

### Field Sample Collection and Processing

Clinical samples used in this study were obtained from patients presenting to clinics run by the Shoklo Malaria Research Unit in Mae Sot, Thailand, Anderson TJ et al. Proceedings of the Royal Society B 2010. Inferred relatedness and heritability in malaria parasites and from a field survey in Chikhwawa, Malawi.

In Malawi, a venous blood sample (5 ml) was collected prior to drug administration from a child aged 47 months presenting to our study site in Chikhwawa with uncomplicated *P. falciparum* malaria (thin smear parasitemia of 1.4% in 2016). The sample was obtained with the parent’s consent as part of a larger study aimed at understanding within-host parasite genetic diversity in malaria patients from an area of intense malaria transmission. The blood sample was collected in an Acid Citrate Dextrose tube (BD, UK), and transported to the laboratory in Blantyre where it was processed as follows: Half of the sample was washed with incomplete RPMI 1640 media and the resulting pellet was mixed with glycerolyte before storage in liquid nitrogen. Parasites used in our FACS experiments were grown from this sample. The other half of the sample was passed through a CF11 column to deplete white blood cells (Venkatesan et al. 2012) and was stored at −80 °C until needed. Ethical approval for the study was granted by the University of Malawi College of Medicine Research and Ethics Committee (Protocol number P.02/13/1528) and the Liverpool School of Tropical Medicine Research Ethics Committee (Protocol number 14.05). The *P. falciparum* laboratory line, HB3, used for optimization of gating and WGA experiments was obtained from MR4 (Manassas, VA), and was maintained in the laboratory for several weeks as needed.

### Sterility Guidelines

Gloves, a face mask and a sterile gown were worn at all times prior to library preparation. Cell sorting materials and MDA reagents were prepared in “PCR Hood 1” which is housed in a “malaria-DNA free” room separate from the main lab whereas thawing of frozen single cells and initiation of the MDA protocol was carried out in PCR Hood 2, behind a floor-to-ceiling plastic barrier. MDA was initiated in the main lab on a dedicated thermocycler, whereas library preparation was performed on a separate thermocycler in another lab. PCR Hood 1 was equipped with standard pipettes and presterile filter tips whereas PCR Hood 2 was equipped with positive displacement pipettes and presterile displacement tips to reduce the possibility of aerosol contamination between samples. All tubes and tube racks were autoclaved (dry vacuum cycle, 30 min) before use. HB3, THB1, and THB2 cells were sorted into PBS (Qiagen), whereas MAW0 cells were sorted into recently autoclaved PBS (Lonza).

Prior to use, the interior of the hood was cleaned by wiping down all pipettes, tube racks, and tabletop centrifuges with a series of solutions: 1% bleach, DNAzap according to manufacturer’s instructions, a 70% ethanol wash, and an optional sterile water wash, followed by 15 min of UV irradiation. The thermocycler and PCR tube cold rack were wiped down with DNAzap and ethanol before use. We elected not to use UV treatment for reagents and reagent tubes, as the recommended exposure (Woyke et al. 2011) yellowed the manufacturer’s PCR tubes. This may introduce unknown byproducts into the reaction or physically stress the tube, which could compromise sterility.

### Sort Preparation

In PCR Hood 1, 5 μl of 1X PBS (Lonza, Accugene, autoclaved) or 1X PBS (Qiagen) was delivered into individual, autoclaved PCR tubes with a repeat pipettor. PBS aliquots were stored on a 96 well plate inside of an autoclaved sleeve until use the following day.

### Cell Staining

HB3 cells were grown to asynchrony or purified red blood cells isolated from patient samples (∼0.2–0.5 ml) were revived and grown (supplementary Text S2, Supplementary Material online) in resealable culture chambers, flushed with gas (5% CO_2_, 5% O_2_, Balance N_2_). After culture, cells were washed once with PBS and centrifuged (425 × g). Five to eight microliters of RBC pellet (∼10^8^ cells, typically) were resuspended in a 1× PBS solution that included 2.5 μl of Vibrant Dye Cycle Green dye (5 ml). The suspension was covered in foil to prevent light exposure and incubated at 37 °C for 30 min with intermittent inversion every 5–10 min. RBCs were washed twice in 10 ml PBS and resuspended in 5–8 ml of PBS and protected from light.

### Cell Sorting

Individual cells were sorted into 0.2 ml PCR tubes (5 μl 1× PBS) held on a 96-tube rack one at a time by MoFlo Astrios (Beckman Coulter). Events were gated according to DNA fluorescence and sorted in single-cell sort mode with a drop envelope of 0.5, with each cell typically taking under 15 s to sort. Captured cells were immediately stored on dry ice and transferred to -80 °C storage.

### Standard PCR

PCR reactions (Takara) included 10 ng of target DNA and 1 μM custom primers (*pfcrt*-L AGGTTCTTGTCTTGGTAAAT and *pfcrt*-R TTTGAATTTCCCTTTTTATT; *dhfr*-F ACGTTTTCGATATTTATGC and *dhfr*-R TCACATTCATATGTACTATTTATTC) using the following program: Hold 94 °C 2 min; 5 cycles 94 °C 0.5 min, 50 °C 0.5 min, 60 °C 0.5 min; 25 cycles 94 °C 0.5 min, 45 °C 0.5 min, 60 °C 0.5 min; Hold 60 °C 2 min; Hold 4 °C, and resolved by standard 1% agarose electrophoresis.

### WGA

Repli-g MDA reagents were thawed, prepared and aliquoted (Qiagen MIDI kit, QIAseq FX single-cell DNA kit) in PCR Hood 1 and transferred to PCR Hood 2. The enzyme mastermix was kept on ice during cell lysing steps. Sorted samples were thawed in PCR Hood 2, spun briefly and the reaction was initiated according to manufacturer’s instructions with the exception of 20 μl of total mastermix added per sample instead of 40 μl for REPLI-g reactions (Text S1). During lysis, tubes were kept outside of the hood on a precooled rack. The reaction proceeded on a thermocycler with heated lid for 4.5 or more hours. We recommend a routine amplification time of 6.5 h. For 24 MAW0 single-cells, the manufacturer’s protocol for QIAseq FX Single-Cell DNA Kit was followed. In both cases, MDA DNA products were recovered from reaction mixtures with Zymo Genomic Cleanup kits and eluted in 55 μl of water. Library preparations (see below) and MDA products are stored long term at -80°C on separate shelves to reduce the potential for contamination.

### Library Preparation

#### KAPA

Illumina sequencing libraries were prepared by the KAPA HyperPlus Kit according to manufacturer’s guidelines using a thermocycler with programmable lid temperature and Agencourt AMPure XP for cleanup and size selection with the following parameters. We used 100 ng of starting DNA material (MDA or bulk extracted DNA), carried out a 1 h ligation of adapters (5 μl of 15 μm Bioo [NextFlex 48 barcode adapters] in 110 μl reaction), and amplified adapter-ligated libraries for 6 cycles.

#### Qiagen

Library preparation was carried out according to manufacturer’s instructions for QIAseq FX single-cell DNA Kit. The recommended standard protocol yielded large, undesired products, so we carried out an additional 1:1 cleanup step followed by an additional size selection step according to the KAPA Hyperplus Kit protocol. Additional experiments revealed improved product distribution by increasing fragmentation incubation time to 33 min before proceeding with the recommended cleanup and size-selection step in the Qiagen protocol.

#### WGS Library Quality Control

The size of each Illumina DNA library was determined by HS DNA chips (Agilent) or DNA Tapestation according to manufacturer’s instructions.

Pooled libraries were generated by multiplexing either 12 or 24 uniquely barcoded libraries and sequenced on a single lane Illumina HiSeq 2500 using 101 bp paired end sequencing with v3 chemistry. Raw sequence reads were demultiplexed and .fastq files generated using bsl2fastq v2.17.

#### Bioinformatics

We aligned each .fastq file to version 3 of the 3D7 reference genome sequence (http://www.plasmodb.org) with BWA-MEM v0.7.5a (Li 2013). PCR duplicates and reads mapping off chromosomal ends were removed with Picard v1.56 (http://broadinstitute.github.io/picard/). We performed base recalibration and realigned around indels using GATK v3.5 (DePristo et al. 2011). Genotypes were called using GenotypeGVCFs in GATK v3.5 using the QualByDepth, FisherStrand, StrandOddsRatio, VariantType, GCContent, and TandemRepeatAnnotator annotations with max_alternate_alleles set to 6. After variant score recalibration we kept all loci with a VQSLOD score >0 and filtered out SNP calls outside of the “core” genome, defined in Miles et al. (2016). For comparative analysis we downsampled bam files to 30× coverage using the -dfrac flag in the GATK engine and calculated coverage statistics using the flagstats and DepthOfCoverage tools. Genomic intervals were subset using Bedtools v2.25.0 (Quinlan 2014).

For identity by descent (IBD) analysis we scored regions of IBD using Beagle v4.1 (Browning and Browning 2013). As this tool was designed for diploid data we generated doubled homozygotes and collapsed together overlapping estimates of IBD. We generated a novel genetic map for this analysis using a collection of genome sequences from clonal Malawian isolates (Nkhoma et al unpublished) using the rhomap function in LDHat v2.2 (Auton and McVean 2007).

All statistical analysis was performed in R v3.3.0 and used the Intervals v0.15.1 (Bourgon 2015), R package version 0.15.1 (https://CRAN.R-project.org/package=intervals), and SeqArray v1.12.9 (Zheng and Gogarten 2016) SeqArray: Big Data Management of Whole-genome Sequence Variant Calls. R package version 1.12.9. (http://github.com/zhengxwen/SeqArray) packages.

## Results

### iRBCs That Contain High DNA Content Are Superior Targets for WGS

**Fig. 1.**
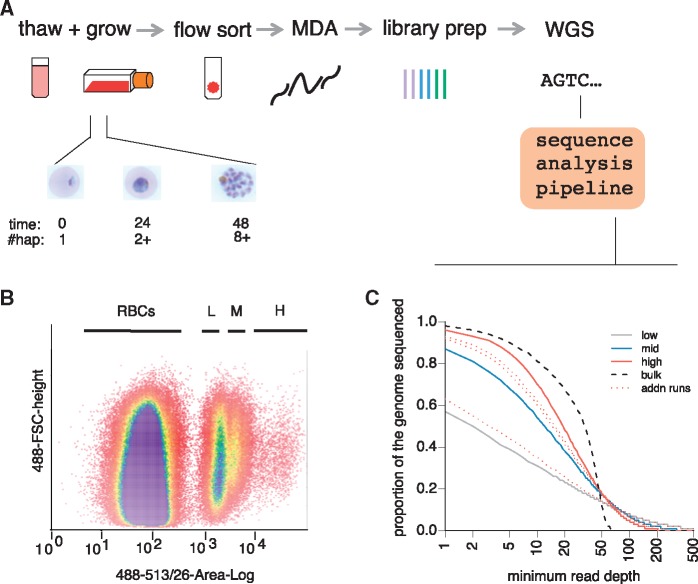
—Targeted single-cell genomics of late-stage malaria parasites. (*A*) Cryopreserved iRBCs are thawed and grown under standard conditions for 40 h, generating late-stage parasites with multiple genome copies. DNA-stained iRBCs are sorted into individual tubes by FACS. To generate high quality reactions, high DNA content, late-stage parasites in the H gate are freeze–thaw lysed prior to MDA, library preparation and WGS. (*B*) An asynchronous culture of HB3 containing parasites with different amounts of DNA. The *x*-axis shows the fluorescence intensity, and the *y*-axis the size of each cell. (*C*) Genome coverage obtained by sequencing cells from the L, M, and H gates. The plot shows the proportion of the genome (*y*-axis) sequenced to at least a given minimum read depth (*x*-axis). The black dashed line is data obtained by routine sequencing of high quality DNA from a laboratory derived line. The solid lines denote cells from the L (grey), M (blue), and H (red) gates, with dotted red lines additional cells from the H gate. All libraries were downsampled to 30× coverage for comparability.

In the 48 h blood stage of the malaria lifecycle, parasites are haploid and in the earliest stage of their life cycle contain only a single copy of the genome. By the latest life cycle stage, they harbor ∼16 copies (Reilly et al. 2007) prior to bursting and invasion of new RBCs. To test whether targeting late-stage iRBCs by FACS (Dekel et al. 2017) would generate gains in genome data quality, we performed WGA of parasites with increasing DNA content. A commonly used laboratory-adapted line, HB3 (MR4, VA), was thawed and cultured for several weeks to allow asynchrony in cell cycle progression. Parasite DNA in the cultured cells was stained with Vybrant DyeCycle Green (fig. 1*A*) and analyzed by FACS. Three fluorescent subpopulations of DNA-containing cells were observed by flow cytometry, reflecting the asynchronicity of the culture. Event gates capturing these three populations were drawn, denoted low (L), medium (M), or high (H), based on increasing levels of fluorescence due to increasing DNA content (fig. 1*B*).

Individual cells from each gate were sorted into single tubes, freeze-thawed, and WGA was carried out by multiple displacement amplification (MDA, REPLI-g Midi, Text S1). Stringent protocols were implemented to minimize the risk of contamination (Materials and Methods, Text S2). As an initial test for DNA quality, two parasite-specific genes, *pfcrt* and *dhfr* were amplified from HB3 WGA reaction products by standard end-point PCR, providing a qualitative assessment for genome amplification. Fifty percent (5/10) of reactions failed to yield product for cells in the L gate, compared with 20% (2/10) for cells in the M gate, and 0% (0/14) for cells in the H gate (supplementary fig. S1, Supplementary Material online). We took this as a preliminary indication that dropout of alleles might occur less readily in MDA of iRBCs containing higher DNA content.

### Near-Complete Capture of Malaria Genomes

We next performed WGS of representative reactions from cells sorted in each gate. Two metrics were used to determine the usefulness of sequencing data: Read purity (the fraction of observed reads that map to the *P. falciparum* reference) and genome coverage (the fraction of the genome with at least one read mapped). For the HB3 cells (three cells total, one cell from each gate), >87% reads mapped to the *P. falciparum* reference genome in every gate, demonstrating that our guidelines for sterility were sufficient to eliminate outside contamination. Interestingly, the HB3 L gate reaction was marked by moderate genome coverage (64.8% coverage) whereas cells sorted by the M (93.6% coverage) and H gates (97.4% coverage) yielded high coverage, similar to the genome coverage recovered from bulk DNA (97.8%; fig. 1*C*, supplementary table S1, Supplementary Material online). Subsequent sequencing of three additional cells from the H gate confirmed high capture of the parasite genome in two out of three cells (95.6%, 95.2%, 71.5%).

Shortening the length of MDA reactions has been shown to improve the evenness of genome coverage by restricting runaway amplification in other contexts (Gole et al. 2013). Thus, we sampled three reactions (L, M, and H gate) across several time-points (4.5, 8, and 16 h) of WGA. Surprisingly, all single-cell reaction times yielded similar depth of coverage (supplementary table S1 and Text S1, Supplementary Material online), suggesting amplification bias is minimal between 4.5 and 16 h of reaction, perhaps due to diminishing enzyme activity or peculiarities of primer annealing in AT-rich genomes.

Previously, single-cell sequencing of malaria cell lines (HB3, 3D7) and clinical samples generated sequence data with variable genome coverage (Nair et al. 2014). Cryopreserved clinical samples are dominated by early stage parasites which both circulate in the bloodstream and survive cryopreservation. Our previous protocol used an overnight (18 h) culture, after which parasites are unlikely to have progressed sufficiently far through the cell cycle to have undergone multiple rounds of DNA replication. We redesigned our protocol to enrich for late-stage parasites by analyzing two clinical samples collected on the Thai-Burmese border, grown either for 18 h or 40 h prior to FACS (THB1, THB2, respectively). Infections characterized by a parasitemia of 0.5–1.5% (as determined by thin-smear on day of collection and FACS on the day of analysis) were chosen for this and subsequent analysis. This fraction of positive events readily generates true positive events well over instrument noise. Analysis of 25 single-cell DNA libraries from these samples showed that cells sorted from the H gate generated better genome data quality than L or M gate cells (supplementary fig. S2 and table S1 and Text S1, Supplementary Material online), consistent with the trend observed for HB3 cells (fig. 1). However, the breadth of genome coverage was lower than for laboratory-derived cells (L gate: mean = 31.5% [range 22–41%], M gate: mean = 50.0% [range 24–80%], H gate: mean = 68.2% [range 54–96%]).

**Fig. 2.**
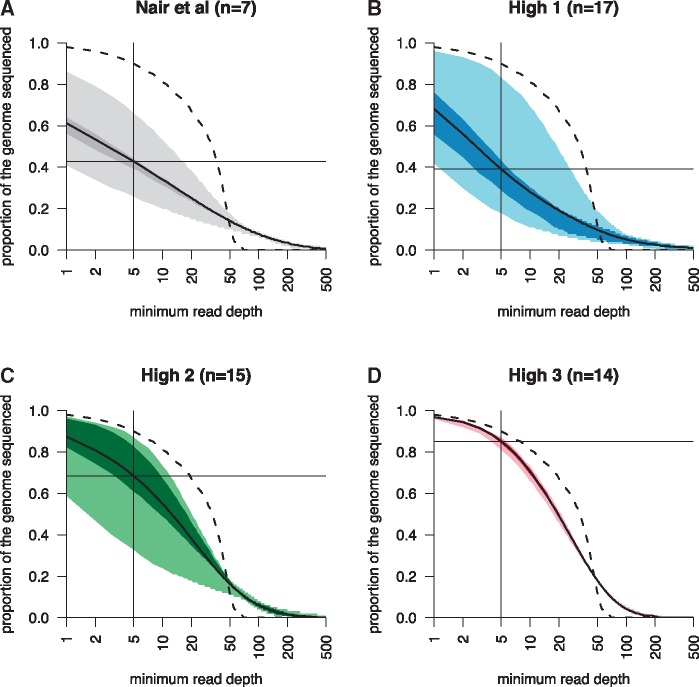
—Comparison of genome coverage for single-cell WGS libraries. Each plot shows the same statistic as in figure 1C, including the median value (solid line) with the interquartile range (dark shading), and the range (light shading). Genome coverage as a function of read depth from WGS data collected by previous work THB0 (*A*) or H gate-sorted cells grown for 40 h from THB2 (*B*), MAW0 (*C*), (*D*). (*A*)–(*C*) were processed by REPLI-g and KAPA HyperPlus library preparation with PCR amplification. (*C*) and (*D*) were sorted into sterilized PBS (Lonza) and (*D*) was processed with the QIAseq FX single-cell DNA Kit. All libraries were downsampled to 30× coverage for comparability.

To adapt our protocol further for clinical samples, we made two additional modifications prior to analyzing a third clinical sample collected in Chikhwawa, Malawi (MAW0, supplementary fig. S3, Supplementary Material online). First, since amplification of small amounts of DNA is extremely sensitive to DNA contamination in reagents (Motley et al. 2014; Suyama and Kawaharasaki 2013), we autoclaved the PBS used to capture sorted cells. Second, we implemented a PCR-free library preparation method to reduce amplification bias. Coverage was uniformly high across the 48 single cells, with a mean of 90.7% (range 52.4–98.6%) of the genome containing at least one correctly mapped read and was not due to inadvertent capture of multiple cells (supplementary fig. S4, Supplementary Material online). This rose to an average of 92.3% (range 48.2–99.8%) of the genome after excluding highly polymorphic regions generally not amenable to most routine sequence analysis (supplementary table S1, Supplementary Material online). Furthermore, PCR-free library amplification improved the mean genome coverage and reduced sample-to-sample variation in genome coverage (fig. 2 and supplementary table S1, Supplementary Material online).

To further ensure that our data did not include the capture of multiple cells, we analyzed the proportion of mixed base calls at high coverage (>30×) sites. This resulted in the exclusion of 5/48 single cell sequences where >5% of sites contained <95% of reads supporting a single genotype. These were likely conservative thresholds as putatively clonal *P. falciparum* genome sequences can frequently contain unfixed base calls due to challenges in aligning to the highly AT-rich and repetitive reference genome (Gardner et al. 2002).

Clear gains in single-cell data quality emerge when comparing the progress of our genome coverage through successive methodological improvements. We took the seven cells (THB0) sequenced using our previous protocol (Nair et al. 2014), and 46 single-cell sequences from the three treatments described here: (i) H gate with manufacturer’s PBS and KAPA HyperPlus with PCR Library Amplification Kit (“High 1”, THB2), (ii) same as *i* except for inclusion of autoclaved PBS sort “capture” buffer (Lonza) (“High 2”, MAW0) or (iii) same as *ii* except with QIAseq FX single-cell DNA Kit (“High 3”, MAW0). We randomly downsampled BAM files so each library had a mean of 30× coverage for comparability. Notably, cells processed by the original method (Nair et al. 2014) had been preselected as containing a high proportion of genotype calls from a larger panel of isolates, potentially overestimating the quality of this data. Figure 2 shows the steady increase in data quality throughout the development of this method. We attribute these improvements to the targeting of late-stage parasites, using high quality, sterile reagents and omitting PCR amplification of library preparations. We have gathered similar single-cell genomic data quality using this approach on other clinical samples, suggesting MAW0 is not a unique case (supplementary fig. S5, Supplementary Material online).

As is the case for human nuclei (Leung et al. 2015), we demonstrate that malaria parasites undergoing DNA replication serve as better starting points for WGA. We hypothesize that the presence of multiple copies of the same template increases the chances that a given DNA segment will be successfully primed and amplified. However, other factors may also play a role in the accessibility of DNA to MDA reaction components, such as protein: DNA contacts and differences in membrane composition at different life cycle stages. These were concerns for malaria genomes, which are housed beneath several membranes and require both freeze–thaw and chemical lysis prior to MDA (Nair et al. 2014). Additional gains in purity and target genome coverage in single-cell WGA might be attained by including malaria-specific primer sets Sundararaman SA et al. Nature Communications 2016. Genomes of cryptic chimpanzee Plasmodium species reveal key evolutionary events leading to human malaria, using exome capture, or optimizing UV treatment of reagents prior to WGA (Woyke et al. 2011), though our observed read purity is sufficiently high for most downstream applications.

The MAW0 sample was collected from Chikhwawa, Malawi where infected individuals are likely to contain many parasite lineages. This presented an excellent opportunity to test whether our optimized protocol could dissect the complexity of a potentially challenging, diverse infection. We examined two features of the data: 1) how well haplotypes from the infection are represented in single-cell genomic analysis, and 2) the overall patterns of diversity and relatedness between parasites.

### Haplotypic Diversity

**Fig. 3.**
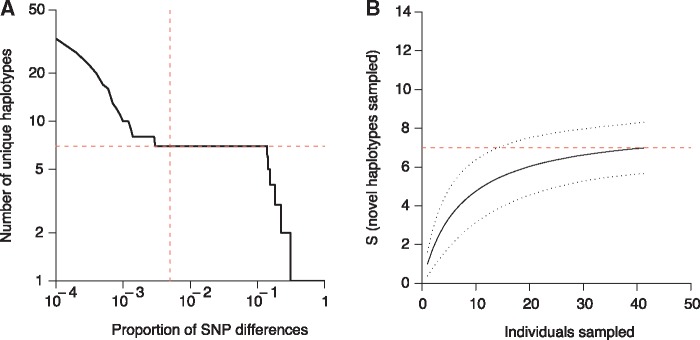
—Estimation of the number of unique haplotypes in a complex infection. (*A*) The number of unique haplotypes observed in MAW0 using an increasingly permissive threshold for pairwise differences. The vertical red line shows the point at which we estimate few errors will define new haplotypes whereas the horizontal red line shows the estimated number of haplotypes at this threshold. (*B*) Rarefaction curve for 43 single cells from the MAW0 infection, 95% confidence interval in dashed black line. The red dashed line is the estimated number of haplotypes from (*A*).

Chikhwawa is an area of intense malaria transmission, where polyclonal infections predominate. The number of unique haplotypes within an infection is a key measure of diversity. Directly counting the number of unique haplotypes is complicated by the accrual of de novo mutations and sequencing errors in individual haplotypes. The number of unique haplotypes estimated in MAW0 rapidly declines over low levels of pairwise SNP differences, reflecting the exclusion of de novo mutations and sequencing and/or amplification-induced errors (fig. 3*A*). Given a suitable estimate of mutation and error rates expected during single-cell sequencing (the error rate of short-read sequencing is ∼1 × 10^−7^ per base, the estimated error rate of WGA is 1.4 × 10^−5^ per base, (de Bourcy et al. 2014), 202 false positive mutations are expected in the 20 Mb core genome sequence (equating to 0.25% of the 19,713 SNPs called in MAW0 data). We suggest that a suitable threshold to collapse together individual sequences into shared haplotypes for MAW0 is 0.5% (0.25% differences per sequence) and shown by the vertical red dashed line in figure 3*A*. Beyond differences of >10% between sequences the estimates rapidly collapse as genuine distinguishing variation is eliminated. This estimate of seven distinct haplotypes is similar to previous estimates from single locus deep sequencing performed in Malawi (Juliano et al. 2010).

In order to determine whether or not the infection had been sampled to an appropriate depth, rarefaction analysis (Colwell et al. 2012) was performed on the haplotype frequencies (fig. 3*B*), using the divergence threshold shown in figure 3*A* (estimating seven haplotypes). On the basis of the data the true number of haplotypes present in this infection may be as high as 8, suggesting we have captured nearly all of the haplotype diversity at this error tolerance. While in the current analysis, we have been conservative in our treatment of de novo mutations and sequencing and/or amplification errors, improvements in laboratory, and bioinformatics tools may allow us to distinguish between these categories in the future.

### Relatedness of Individual Parasites

In addition to providing estimates of the number of distinct haplotypes in an infection, single-cell sequencing can provide details on the patterns of diversity and relatedness contained within each haplotype. We used two common approaches to estimate relatedness between individuals to illustrate this: Pairwise allele sharing and identity-by-descent (IBD). From molecular data, the relatedness of individual parasites can be understood through analysis of sequence identity (identity-by-state) as well as by contiguous segments of DNA shared between parasites, IBD. Comparisons of IBD between individuals can indirectly determine their relatedness within a given population (Gusev et al. 2012; Ralph and Coop 2013). The lower the number of meioses separating two haplotypes is, the longer these blocks of IBD will be. Thus, more closely related parasites will share more, and longer, blocks of IBD than unrelated parasites. This process is closely correlated with the proportion of alleles two individuals share. Importantly, unlike other methodologies, estimations of allele sharing by IBD are not complicated by obstacles such as missing data and variable recombination rates.

**Fig. 4.**
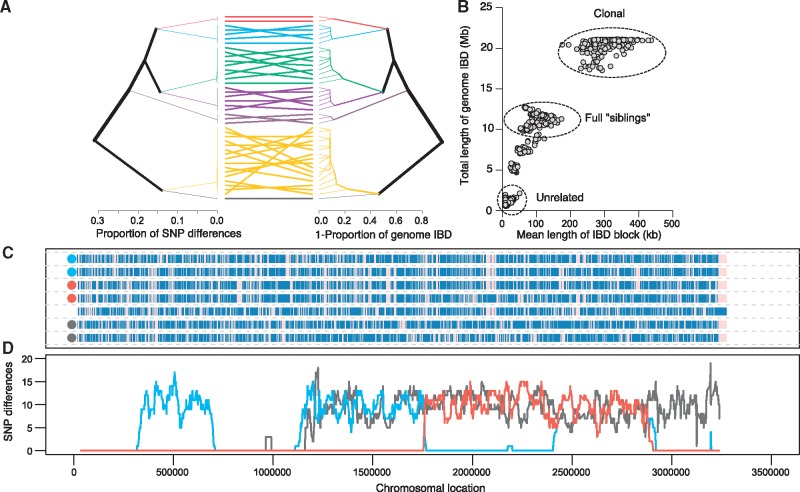
—Relatedness of individual parasites. (*A*) UPGMA tree of pairwise allele sharing (left) and proportion of genome IBD between individual parasites (right) in the MAW0 infection. The haplotypes inferred in figure 3*A* are shown in matching colors in the lines joining the tree branches. (*B*) Relationship between total fraction of IBD and IBD length between parasites. Parasites from identical haplotype groups shared IBD across nearly the entire genome (dots in the upper right), conversely parasites from the most distantly separated haplotype groups (i.e., red vs. dark grey) shared near zero IBD (dots in the bottom left). (*C*) SNP map of chromosome 14 for the 7 consensus haplotypes. (*D*) The number of SNP differences in 20 SNP windows in pairwise comparisons between haplotypes. The haplotypes compared for each colored line are denoted by dots in (*C*).

Figure 4*A* illustrates the proportion of pairwise differences with a UPGMA tree, where highly related individuals cluster together and clone frequencies range from 2.4% to 38.1%. On the basis of the threshold suggested above (0.5% SNP differences) seven unique haplotypes were detected. IBD analysis is broadly concordant with pairwise allele sharing, showing seven distinct clusters. The mean length and total length of IBD within an infection track closely with pairwise allele sharing, with comparisons between haplotypes with greater numbers of SNP differences also showing smaller blocks of IBD with lower levels of genome-wide IBD (fig. 4*A*). Several classes of relatedness emerge, including those concordant with pairs of full “siblings” and unrelated parasites (fig. 4*B*). Consensus haplotypes from each cluster allowed direct phasing of 18,643 of 19,713 SNPs (94.6%) which are unfixed in this infection (fig. 4*C* and *D*). The genetic architecture of MAW0 appears to be very similar to what has been seen in polyclonal infections previously collected in Malawi and Thailand (Nair et al. 2014; Nkhoma et al. 2012).

### Single-Cell Genomics Accurately Captures Allele and Haplotype Frequency in Polyclonal Infections

**Fig. 5.**
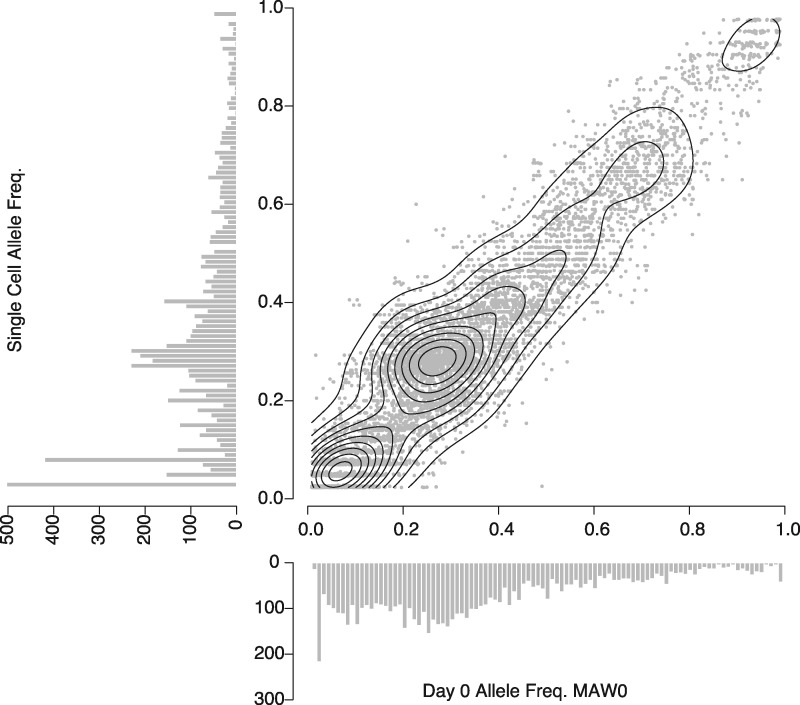
—Frequency of alleles detected in bulk DNA at time of thaw and pooled single-cell library data. 9,766 unfixed sites with a read depth of at least 50× in the bulk sample, and had genotype calls for 80% of the single cell sequences were used to estimate sampling bias. A histogram showing the raw counts for each group is attached to the relevant axis. A contour map is overlaid the scatterplot to highlight the density of points lying along the diagonal.

This new genome capture strategy includes both extending the time of culture and targeting cells with high DNA content by flow cytometry. Since these actions could place artificial restrictions on which haplotypes are surveyed, it is important to determine whether the single-cell genomes recovered by this method are representative of the diversity found in the original infection. To address this, bulk DNA, which should contain nearly all diversity present at the time of collection, was extracted from a frozen red blood cell preparation of MAW0 and deep sequenced to provide a robust estimation of within-host allele frequencies. We then compared the allele frequency of 9,766 sites between the bulk sample and computationally pooled DNA of 43 out of 48 single-cell genomes passing quality control filtering (fig. 5). There is high correlation between the data sets (*r*^2^ = 0.96) suggesting minimal bias introduced by sampling and/or cell culture.

**fig. 6.**
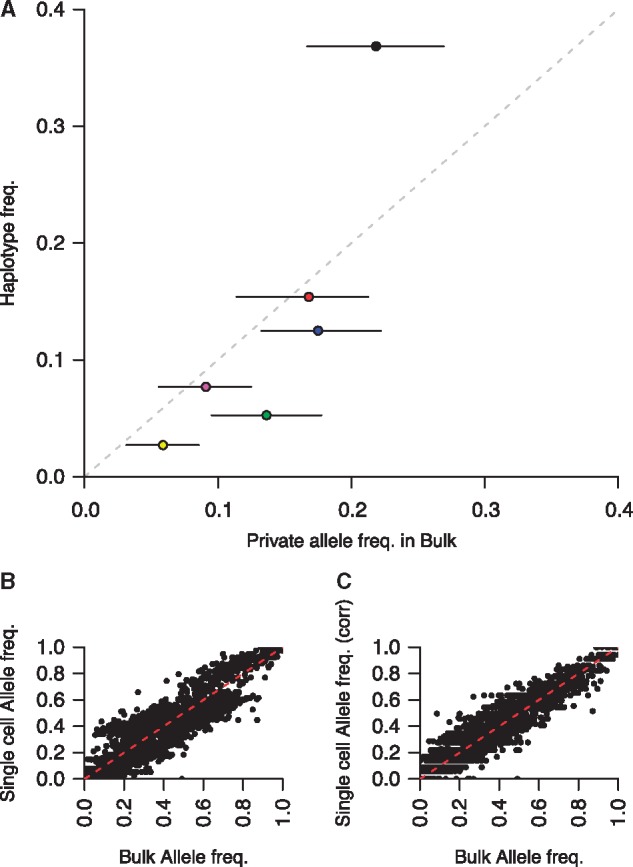
—Unique mutations from single-cell sequencing can be used to infer haplotype abundance in bulk genome sequence. (*A*) Unique mutations from each haplotype group were used to estimate the bias in estimating their abundance in the single-cell sampling. The interquartile range of the allele frequencies for each haplotype is shown by black bars surrounding each plot. These unique allele frequencies were used to correct the haplotype abundances. The original comparison between bulk and single cell allele frequencies ((*B*); a replicate of fig. 5) and (*C*) the corrected data.

Another way to estimate the sampling bias of single-cell sequencing is to estimate the frequency of each *haplotype* in the bulk sequence data. We can easily determine the prevalence of haplotypes by identifying mutations that are unique to each of the haplotypes. In total 4,375 SNPs were unique to a single haplotype, with a mean of 625 unique SNPs per haplotype. The frequency of each unique SNP and the haplotype it is derived from is shown in figure 6. Given this data it was also feasible to correct the abundance of each haplotype in the patient. One haplotype lacked any private mutations, as such its abundance was estimated as the remaining unexplained haplotype frequency (the other inferred haplotype frequencies sum to 0.804). This resulted in a modest improvement in correlation between bulk and single-cell allele frequencies to *r*^2^ = 0.98). Figure 6 shows our method captures the vast majority of diversity within a complex infection. These results suggest culture conditions are not exceedingly restrictive for the survival of parasites in this method, which requires cells to develop into the late-stages for analysis. Other factors, such as unreported drug administration, or lasting effects of the immune system could impair parasite growth in culture. It is possible outliers that are missed by our approach could be detected by additional sampling of individual cells or patient aliquots, though probabilistically, returns are diminishing (fig. 3*B*) and this may be cost-prohibitive.

## Discussion

A major concern for single-cell genomics is the accurate capture of haplotype diversity and frequency in the original sample. For the complex infection analyzed here, these metrics were maintained. Though this sample suggests 40 h of culture does not introduce substantial bias, we recommend inclusion of bulk DNA captured at time point zero (directly from the patient arm) for all single-cell genomics analyses as a critical control.

That late-stage parasites, cultured prior to re-invasion may closely capture the abundance of haplotypes found in the original infection is encouraging for future studies. We anticipate this method may be adaptable to the single-cell genome analysis of *Plasmodium vivax*, which cannot currently be cultured for multiple invasion cycles. Additionally, low-parasitemia infections, which have very small fractions of iRBCs, would be poor samples for flow cytometry due to the likelihood of high false positive rates caused by extended sort times (supplementary Text S1, Supplementary Material online). However, it is possible that magnetic enrichment of late-stage parasites could be performed prior to sorting such that these unknown malaria haplotypes may be individually studied.

Targeted isolation of late-stage malaria parasites allows efficient, detailed reconstruction of polyclonal malaria infections at the single-cell level. This strategy recovered nearly complete haplotypes in nearly every sample tested, bypassing the need to determine successful WGA prior to WGS by costly quality control. Thus, rapid and cost-effective single-cell sequencing is accessible to most laboratories. Future single-cell genomics approaches may benefit from the strategy of targeting multinucleated cells, especially for Apicomplexan parasites. We anticipate that large scale examination of malaria infections at single cell resolution will yield exciting new insights into the fine scale structure of parasite populations.

## Supplementary Material

Supplementary data are available at *Genome Biology and Evolution* online.

## Supplementary Material

Supplementary Materials
