# Supplementary material for: High-Resolution Single-Cell Sequencing of Malaria Parasites
Source: Genome Biol Evol. 2017 Dec 6;9(12):3373–83. doi: 10.1093/gbe/evx256 (PMC5737330; doi:10.1093/gbe/evx256)
Supplement: Supplementary Materials [file evx256_supp.docx]

**Supplementary Material**

**Supplementary Table**

Supplementary Table 1. Coverage statistics for all single-cell sequencing experiments

| Sample  (culture time) | Treatment  Name | Gate | Kit | PBS  sterile  Lonza | Read Depth  *mean [range]* | Read Purity  *mean [range]* | Genome Coverage  *mean [range]* | n >80% purity / n sampled |
| --- | --- | --- | --- | --- | --- | --- | --- | --- |
| THB1  (18 hr) |  | L | REPLI-g  KAPA HyperPlus w/ PCR |  | 25.84  [13.4-45.38] | 91.3  [80.18-95.95] | 23.41  [11.83-41.8] | 7/10 |
|  |  | M+H | REPLI-g  KAPA HyperPlus w/ PCR |  | 67.56  [31.36-120.50] | 97.16  [96.08-98.64] | 61.56  [28.92-108.64] | 3/10 |
| THB2  (40 hr) |  | L | REPLI-g  KAPA HyperPlus w/ PCR |  | 30.32  [25.59-33.82] | 97.39  [94.58-99.45] | 27.77  [23.45-31.19] | 3/3 |
|  |  | M | REPLI-g  KAPA HyperPlus w/ PCR |  | 40.52  [38.16-42.46] | 97.17  [94.89-98.45] | 37.01  [35.06-38.81] | 4/4 |
|  | “High 1” | H | REPLI-g  KAPA HyperPlus w/ PCR |  | 32.02  [0.01-84.21] | 92.20  [80.42-99.42] | 29.54  [0.01-67.66] | 47/54 |
| MAW0  (40 hr) | “High 2” | H | REPLI-g  KAPA HyperPlus w/ PCR | x | 39.13  [15.64-67.91] | 96.1  [93.56-98.21] | 36.02  [13.77-73.11] | 24/24 |
|  | “High 3” | H | QIAseq FX single-cell DNA Kit | x | 43.37  [7.31-145.31] | 91.26  [80.29-97.19] | 38.13  [6.41-127.97] | 24/24 |

**Supplementary Figure Legends**

Fig. S1 Success rate of *dhfr* and *pfcrt* amplification from L, M, and H gate MDA products. Representative gel for pfcrt end-point PCR product negative control with no DNA (-), HB3 bulk DNA 10 ng (+), MDA product (10 ng) from single-cells captured in L, M, or H gates (L, M, H, respectively) (left). Summary table of successful end-point PCR reactions for each gate and PCR gene product (right).

Fig. S2 Genome coverage for single-cells collected from the L, M, H gate in two clinical samples (THB1 and THB2). THB1 “M+H” reactions are plotted as “Mid-gate”, as H gate events were infrequent in this sample (see Text S1).

Fig. S3 Flow cytometry plot of MAW0. 48 positive events in the H gate were sorted for the single-cell genomics workflow, omitting post-MDA quality control measures.

Fig. S4 Low rate of contamination in single-cell sequencing data. The proportion of sites with <95% of reads showing a single genotype call. Sites were filtered to only those likely to be informative (though with a read depth of >30X). We excluded samples with >5% unfixed sites, retaining 43 sequences for downstream analysis. Five putatively clonal clinical samples are shown for comparison on the left side of the plot, and the bulk DNA sequence from MAW0.

Fig. S5 Genome coverage for single-cells collected from the H gate in three Malawi clinical samples (MAW0, MAW1, and MAW2). The median value (solid line) with the interquartile range (dark shading), and the range (light shading) of single-cell genome coverage for three patient samples (H gate-sorted cells). Parasite-infected RBCs were grown for 40 hours and sorted into sterilized PBS (Lonza). WGA and sequencing libraries were prepared with the QIAseq FX single-cell DNA Kit. Only cells characterized by 30X coverage or better are included for comparability. For MAW1 and MAW2 the median number of bases with >=1 read mapped is 97.3% (range=95.6%-98.4%) and 96.6% (range=94.0%-97.9%).

**Supplementary Figures**

Fig. S1

Fig. S2

Fig. S3

Fig. S4

Fig. S5

**Supplementary Text**

**Text S1**

*Repli-g MDA optimization*

Lowering the reaction time of MDA has been shown to improve genome coverage, likely by restricting the time available for runaway amplification of any given loci to occur. We hypothesized that similar results might be achieved for malaria DNA and sampled single-cell MDA reactions at reaction times of 4.5, 8, or 16 hours prior to deep sequencing. Unexpectedly, we observed similar genome coverage between all reaction times, suggesting these effects may occur reactions times lower than tested. Additionally, we speculate that bias may be reduced in the *P. falciparum* genome due to the prevalence of AT basepairs. One possible mechanism preventing runaway amplification may involve the debranching and re-priming of low-complexity, low-melting point sequences.

In all cases, single-cell MDA REPLI-g reactions generate moderate yields of amplified DNA product (typically ~0.5-1 μg). With medium-throughput application in mind, we saw a potential opportunity to cut reagent costs by lowering the amount of reaction cocktail used in each sample. In a small comparison of individually-sorted H gate cells (n=2 per sample dilution) similar genome coverage was seen whether using 1X or 0.5X (0.5 - 1.5 μg total yield), but not 0.25X (not detected, <25 ng) of the manufacturer’s recommended reaction cocktail. Thus, subsequent work was carried out using half of the recommended mastermix reaction buffer.

Moving forward, we recommend preparing libraries using QIAseq FX Single Cell Library Kit, which includes MDA and library preparation together, as genomic data quality was higher for samples processed this way.

*Clinical sample sort optimization*

After the initial HB3 experiments, we prepared a clinical sample collected on the Thai-Burmese border using the original protocol to observe whether similar results could be captured from the L, M, and H gates. However, after only 18 hours in culture, a low density of positive events outside of the L gate was observed. This is expected, since the culture time is not long enough to allow for progression to later stages of the cell cycle. Thus, only two flow cytometry gates were collected: L as well as the M and H gates combined. We additionally did not pre-screen samples for quality prior to library amplification. In this experiment, 7 of 10 tested cells in the M and H combined gate had 6% or less of reads map to the reference genome (Table S1), while 7 of 10 cells from the L gate had >=80% reads map. Thus, L gate sorted cells had a much lower chance of environmental contamination than the M and H combined gate. Since early-stage cells dominated the culture at that time point, events in the M and H gates were infrequent and took longer to sort. We hypothesize that the increased time for which the tube was exposed to ambient air increased the likelihood of contamination. Alternatively, observed events in the M and H combined gates could have included low-frequency false positive machine artifacts. Additionally, the mean genome coverage of the 3 successful library preparations sorted by the M and H combined gate was higher than the genome coverage observed by 7 L gate-sorted cells, consistent with the trend observed in HB3.

We reasoned it may be possible to increase the frequency of events in the M and H gates by growing the samples for 40 hours, instead of for 18 hours. Indeed, for a second sample collected in the same region (THB2) and grown for this extended period, events were more dense in the M and H gate, which reduced the time to sort individual cells. For this and other experiments, successful events were generally sorted in <15 s per sample, though we note the window for avoiding contamination may vary substantially from lab to lab. In all cases for THB2, read purity was >94% in all gates. Furthermore, we observed a trend in improved genome coverage similar to HB3 data, where H gate cells generated roughly twice as much coverage as L or M gate cells (Table S1).

Finally, the genome coverage observed for cells in the THB2 H gate is less than what is observed for MAW0. Between these two experiments, we switched from using the manufacturer’s PBS to in-house autoclaved AccuGENE 1X PBS (Lonza) for the sort capture buffer (5 μl in a single 0.2 mL PCR tube). These and additional experiments suggest that using recently autoclaved AccuGENE 1X PBS (Lonza) in place of the PBS provided by Qiagen may contribute to increased quality metrics.

**Text S2**

**PROTOCOL**

REAGENTS & SOLUTIONS

Vibrant Dye Cycle Green (#V35004)

Incomplete cell media (ICM)- 500 mL RPMI 1640 (Gibco #11875119), 12.5 mL HEPES (Gibco #15630-080), 1 mL Gentamicin 10 mg/mL (Gibco #15719-064)

Complete cell media (CM), ICM 313 mL , 25 g AlbumaxII (Thermo #11021-029), 0.156g Hypoxanthine (Sigma #H936)

10X PBS (Ambion #AM9624)

NaCl solutions, NaCl (Sigma #S-7653) in sterile water (Gibco #15230162), filtered by 500 mL filter system (Corning #430770)

Culture flasks (Corning #430168)

AccuGENE 1X PBS (Lonza #51225)

DNAZap (Thermo Scientific #AM9890)

Bleach (Essendant #KIKBLEACH6)

Sterile water (Gibco #15230196)

Nalgene PETG erlenmeyer flasks (Thermo Scientific #41120250)

Free-Standing Microcentrifuge Tubes with Screw Caps (Fisherbrand #02-682-558)

REPLI-g Midi kit (Qiagen #150045 )

KAPA Hyperplus Library Kit with library amplification (KAPA #KK8514)

Bioo NEXTflex DNA Barcodes (#514104)

QIAseq FX single-cell RNA library kit (Qiagen #180733)

Agencourt AMPure XP (Beckman #A63882)

80% ethanol (Fisher Bioreagents)

Glycerolyte (Fenwal #4A7831)

NEXTflex 48 barcodes (Bioo #514104)

Genomic DNA Clean & Concentrator-10 (Zymo #D4010)

EQUIPMENT

PCR Workstation (Airclean 600) “PCR HOOD #1” & “PCR HOOD #2”

Tabletop LSE microcentrifuge (Corning #6765)

Thermocycler 1 (GeneAmp PCR System 9700 Thermo)

Thermocycler 2 (PTC-200 MJ Reseearch)

Incubator Culture Chamber (C.B.S. # M-624)

PCR cold-rack (Eppendorf #022510509)

Microman M10 positive displacement pipette (#F148501G )

Microman M100 positive displacement pipette (#F148504G)

Gilson CP10ST Microman tips (#F148413G)

Gilson CP100ST Microman tips (#F148415G)

Finnpipette Novus Electronic Single-Channel Pipette (Thermo Scientific #9400250)

PCR tubes (Phenix Research #MPX-200)

Sterilization Pouches (Fisherbrand #01-812-54)

Magnetic Stand (Ambion #AM10027)

Cardinal Health Secure-Gard Cone Mask (Dupont Personal Protection #AT7509)

Sterile gowns (Kimberly-Clark #90042)

FILTER HOOD CLEANING PROCEDURE

Don sterile gowns, new gloves, and cone mask.

Prepare 1% bleach in sterile water.

Using a dropper, wet sterile paper towel (included in sterile gown package) with 1% bleach and wipe internal surfaces of PCR HOOD. Dry surfaces with a clean paper towel.

Use DNAzap according to manufacturer’s instructions, including all surfaces and pipettes.

Spray and wipe surfaces with 70% ethanol.

Wipe surfaces with sterile water.

Turn UV light on for 15 minutes.

Unwrap sterile tips and PETG flask (for waste) in hood without touching any surfaces.

Wipe the outside surface of all reagents with 70% ethanol prior to use in the hood.

CELL CULTURE

Thaw purified RBCs at 37^0^C for 1-2 minutes.

Add 12% NaCl (1/5th volume of RBC) dropwise while swirling sample. Let stand for 5 minutes.

Add 1.8% NaCl (5 mL) dropwise while swirling sample. Let stand for 2 min.

Add 0.9% NaCl (5 mL) dropwise while swirling sample. Let stand for 2 min.

Wash cells once in 10 mL ICM, using centrifugation at 425 x g for 5 min.

Add 8 mL CM grow in sealed box flushed with 5% CO2 5% O2, balance N2 at 37^0^C for 40 hr.

SORT TUBE PREPARATION

In PCR HOOD #1, seal approximately two hundred 0.2 mL PCR tubes, two PCR tube plates and several aliquots of 1X PBS (Lonza) in sterilization pouches.

Autoclave on dry vacuum program (30 minutes).

Clean PCR HOOD#1.

Unwrap sterile PETG flask and pre-sterile 200 μl filter tips.

Wipe outside of post-autoclave sterilization pouches with 70% ethanol.

Dispense 5 μl of autoclaved PBS into individual 0.2 mL PCR tubes using a repeat pipettor.

Store tubes at RT overnight on racks, with each rack protected in its sterilization pouch (opened but folded over to prevent air flow).

STAINING

Wash culture 1X times in PBS by centrifugation (425 x g)

Freeze aliquot of resultant pellet for bulk DNA library preparation (typically 100 μl of pellet).

Add 7-8 μL of pellet to 5 mL staining buffer (1X PBS, 2.5 μL Vibrant DyeCycle Green). Protect tube from light with foil.

Incubate at 37^0^C with intermittent inversion every 5 minutes for 30 minutes.

Wash cells twice in 1X PBS.

Resuspend pellet in 5-8 mL 1X PBS.

MDA + LIBRARY PREPARATION (*preferred method*) Qiagen

Clean PCR HOOD #2. Cool PCR cold-rack on ice next to PCR HOOD #2.

Clean thermocycler with DNAzap and 70% ethanol. Stock PETG flask for waste.

Thaw MDA reagents in PCR HOOD #2. We routinely processed 24 reactions at once.

Place MasterMix on ice near PCR HOOD #2, place D2 and Stop solutions in PCR HOOD #2.

Thaw captured cells at RT, pulse on tabletop centrifuge inside of PCR Hood #2.

Use the PCR cold-rack outside of PCR HOOD #2 for “on ice” incubations, to minimize contact of the tube with ice.

Follow the QIAseq FX single-cell DNA Kit manufacturer’s instructions for MDA. Elute DNA with 55 μl dH2O.

Quantify DNA with Qubit BR Assay kit (#Q32850), according to manufacturer’s instructions

*Clean-up*

Purify MDA DNA products with Genomic DNA Clean & Concentrator-10 (according to manufacturer’s instructions), using 14,000 x g for centrifugation steps. After elution, reapply eluant to column for a subsequent elution to increase DNA recovery.

*Library Prep*

Follow the QIAseq FX single-cell DNA Kit manufacturer’s instructions for PCR-free library preparation. We included the optional enhancer reagent. Elute DNA with 38 μl of dH2O. Typical concentrations of prepped libraries were 3-8 ng/μl. Additional experiments revealed improved product distribution by increasing fragmentation incubation time to 33 minutes before proceeding with the recommended cleanup and size-selection step in the Qiagen protocol.

WHOLE GENOME AMPLIFICATION (*intermediate method*) (REPLI-G, Qiagen)

Follow manufacturer’s guidelines with the following additions:

*Preparation*

Autoclave 2 mL tubes (3).

Clean PCR HOOD #1 and #2. Cool PCR cold-rack on ice next to PCR HOOD #2. Clean thermocycler with DNAzap and 70% ethanol.

Prepare D2, MasterMix, and aliquot Stop stocks in PCR HOOD #1. MasterMix was prepared at half volume. We routinely processed 24 reactions at once.

Place MasterMix on ice near PCR HOOD #2, place D2 and Stop solutions in PCR HOOD #2.

Thaw captured cells at RT, pulse on tabletop centrifuge inside of PCR Hood #2.

Follow Qiagen REPLI-g protocol, using the PCR cold-rack outside of PCR HOOD #2 for “on ice” incubations, to minimize contact of the tube with ice.

Deliver half of the recommended volume of MasterMix per sample (20 μl).

Incubate at 30^0^C for 6.5 hours, followed by a 3 min incubation at 65^0^C for reaction inhibition. Can hold overnight at 4^0^C.

WGS LIBRARY PREPARATION (*intermediate method, continued*)

KAPA

Follow the KAPA Hyperplus Library Amplification Kit manufacturer’s instructions with the following parameters:

Initiate each reaction with 100 ng purified MDA DNA.

Carry fragmentation out for 25 minutes.

Use 5 μl of 15 μM illumina-compatible adapters (Bioo).

Use 6 cycles total for PCR amplification.

Elute DNA with 38 μl of dH2O. Typical concentrations of prepped libraries were 5-10 ng/μl.
